# A protocol for clinical trial study of the effect of core stabilization exercises on spine kinematics during gait with and without load in patients with non-specific chronic low back pain

**DOI:** 10.1186/s12998-017-0162-y

**Published:** 2017-11-16

**Authors:** Rasool Bagheri, Ismail Ebrahimi Takamjani, Mahdi Dadgoo, Javad Sarrafzadeh, Amir Ahmadi, Mohammad Reza Pourahmadi, Amir-Salar Jafarpisheh

**Affiliations:** 1 Department of Physiotherapy, School of Rehabilitation Sciences, Iran University of Medical Sciences and Health Services, Tehran, Iran; 2 Department of Ergonomics, University of Social Welfare and Rehabilitation Sciences, Tehran, Iran; 3 School of Rehabilitation Sciences, Nezam St. Shah Nazari Ave. Madar Sq. Mirdamad Biv, P.O Box: 4391-15875, Tehran, Iran

**Keywords:** None-specific chronic low back pain, Core stabilization exercise, Spine kinematic

## Abstract

**Background:**

Non-specific chronic low back pain (NCLBP) is a major public health and global socioeconomic burden with a variety of symptoms such as gait abnormality. Trunk stiffness and deep trunk muscles dysfunction known as guarding mechanism in gait are factors leading to abnormal movement pattern of the spine. Anterior load carriage task during gait is also challenged the trunk stability and its movement pattern. It will be therefore of interest to examine the effect of a Core Stabilization Training Program (CSTP) on the trunk and pelvis kinematics including variability and peak displacement during gait with and without load in NCLBP patients.

**Methods:**

Patients with NCLBP will participate in a program containing 16 sessions of CSTP and perceived pain, disability and kinematic will be evaluated with 100 mm visual analog scale (VAS), Oswestry Disability Index (ODI) and motion analyzing system respectively before and after the intervention. Participants will be asked to walk with self-selected comfortable speed for 3 times without load and 3 times with caring a load with hands.

**Discussions:**

We will quantify the effectiveness of CSTP on the kinematic of trunk, lumbar and pelvis during gait. Comparing the kinematic pattern and movement variability using CV_o_ and CV_p_ can contribute to better understand the motor control strategy and movement pattern of the spine during an anterior load carriage task between patients with NCLBP and healthy.

**Trial registration:**

IRCT number: IRCT2016080829264N1; pre-result.

## Background

Chronic Low back pain (CLBP) is one of the most prevalent problems up to 84% [1, 2]. Despite its prevalence, no specific causes can be found for almost 85% of the CLBP cases [3]. Therefore, this type of low back pain, named non-specific chronic low back pain (NCLBP), acknowledged multifactorial conditions with various dysfunctions, such as gait abnormality [4–6]. Gait is an activity that is repeated frequently throughout the day. The trunk and lumbar spine, however help drive in human bipedal gait [7]. It has been suggested that deficiencies in motor control during gait following LBP produce repeated and prolonged stresses on the spine [6, 8]. Various studies have reported that patients with CLBP alter their movement pattern and control strategy to avoid painful motion [9, 10]. In a recent review, authors have reported that it is unlikely that gait is independently causative of LBP [11], thus the gait observed in CLBP might be considered a symptom instead [6, 12].

In quantitative analysis of spine movement, the peak displacement, velocity and, acceleration as an angular measurement, and the variability of movements have been measured upon three planes including sagittal, frontal and transverse during gait. The movement variability is more affected by 1) the reliability and errors of measurement, 2) repeatability of the motor performance [13]. It also contains information about how movement changes overtime [14]. Gait studies in healthy subjects had shown relatively low variability of movement in the lumbar spine during treadmill walking [15]. Also, people with CLBP displayed less lumbar displacement [6, 8] and higher lumbar movement variability during treadmill walking [6]. CLBP also exhibited more frontal plane coordination variability and more rigid transverse plane coordination variability of the thorax – pelvis compared to healthy in treadmill walking [9]. However, more rigid sagittal and transverse planes coordination variability obtained from studies of CLBP in over-ground walking compared to healthy subjects [16–18]. Therefore, subjects with CLBP exhibit more in phase trunk – pelvis movement coordination and lower repeatability of lumbar spine movement patterns during gait, revealed higher noise and error in the motor performance that supports the “guarded gait” mechanism [10, 16–20]. Gombatto et al. also evaluated the lumbar spine kinematics including displacement in the different movement-based subgroups of CLBP. Although people with CLBP displayed significantly less overall lumbar rotation than controls, they did not show any significant differences in lumbar kinematics during gait among subgroups [8].

Anterior load carriage during walking is frequently the main task in some industries and individuals in their daily activities that repeated throughout the life challenging the spinal stability and trunk-pelvis movement pattern [21]. It has been suggested that loading conditions with high repetition and frequency can cause to muscular hyper-activity and possible tissue failure and pain in lumbar spine [22, 23]. Anterior load carriage causes to increase of the trunk muscle activity and compressive load on the spine that is reported to be a factor of abnormal movement pattern and can cause to development of LBP [21, 24]. Transverse trunk and pelvis coordination is essential and can be affected during gait-including anterior load carriage in various speeds [21]. Evidence demonstrated that patients with CLBP who have reduced stability exhibited more anti-phase trunk and pelvis coordination pattern during gait associated with an anterior load carriage compared to healthy subjects [21].

Previous studies have demonstrated that an association exists between CLBP and dysfunction in the deep trunk muscle activation pattern [25, 26]. The deep muscles such as transverse abdominis (TrA) and multifidus have large contributions to control the intervertebral movement via the proper timing and coordinated activity during a task irrespective to the direction of force [26–29]. There is evidence that the TrA exhibits a reduced amplitude and delay in activation during repetitive trunk and limb movement such as gait [26, 27]. Multifidus and deep paraspinal muscles show similar changes in activity during functional task as well as loading activity in CLBP [26, 29]. It appears that the abnormal kinematic pattern seen in CLBP during gait combined with increased superficial muscles activity such as erector spinae and rectus abdominis [19], contributing to a poor motor control. Other studies revealed an increased uncoordinated activity of lumbar multifidus, longissimus and lateral abdominal muscles [28, 30, 31] supporting the guarding mechanism during gait. Although, the increase in the superficial trunk muscle activity can prevent lumbar spine buckling and increases the lumbar spine stiffness [28], it is also associated with increased compressive loading of the spine which has been considered as a risk factor for spinal pain and degeneration [28, 32].

According to the European guidelines for the management of CLBP, exercise therapy has strong evidence for the reduction of pain and disability and return to work [2]. However, there is no evidence favoring one form of exercise therapy including “stabilization exercise” for the treatment of LBP in the current guidelines. Local muscle training (core training) has developed as an exercise regimen focusing on motor control and muscle capacity can reduce the pain and disability, it also improves lumbo-pelvic stability (LPS) and restores the function of the spine in daily tasks in CLBP [27, 28, 33–36]. These studies recommended that core training exercises focusing on the TrA and multifidus muscles can restore the LPS and can help to recovery from injury enhancing the spine (the lumbar and thoracic) and pelvis as well as the lower limb performance in static and dynamic functional tasks [26, 27, 37]. Based on previous studies, the size of multifidus and TrA muscles is recovered during tasks including the active straight leg rise, abdominal drawing maneuver, hook lying and standing with a load after a period of core training [38–40]. It is also improved the electromyographic activity of these muscles during flexion-extension task [41, 42], lumbar lordosis [43] and motor cortex representation area of the TrA and multifidus in the brain [44]. Data about the effect of exercise therapy on spine kinematics during gait is very limited. However, Steele et al. evaluated the effect of isolated lumbar extension exercise on lumbar kinematics during gait in CLBP [45]. They reported that the sagittal plane pattern variability decreased after this program. However, the mean displacement in three planes and the pattern variability in the transverse and frontal planes remained unaltered after the isolated lumbar extension exercise [45]. Carpes et al. in a pilot study investigated the effect of a program of strength and endurance training on the spine kinematics. They reported that the trunk rotation and right pelvic tilt increased and the lumbar lordosis decreased after this program [43].

It is possible that the trunk stiffness, and altered lumbar and trunk kinematics including higher movement variability and lower displacement are a manifestation of commonly associated core muscles dysfunction [9]. Core muscles dysfunction can contribute to the poor motor control during gait that may result in compensatory trunk stiffness [19]. To the best of authors’ knowledge, the effect of isolated core training program on the between-trial variability and displacement of the trunk has not been investigated yet. On the other hand, the effect of an anterior load carriage task that is disruptive for LPS to alter the trunk movement pattern has not evaluated in previous studies. Thereby, previous studies have failed to reveal the effect of specific core training program on the lumbar, and trunk kinematics including between-trial variability as well as peak displacement during gait including anterior load carriage.

The study hypotheses are as follows:

1. NCLBP group shows a lower between-trial variability and peak displacement of the trunk movement relative to the pelvis in the sagittal, frontal and transverse planes during gait with and without load compared to healthy.
2. NCLBP group shows a lower between-trial variability and peak displacement of the lumbar movement relative to the pelvis in the sagittal, frontal and transverse planes during gait with and without load compared to healthy.
3. Core stabilization exercise therapy increases the between-trial variability and peak displacement of the trunk relative to the pelvis in the sagittal, frontal and transverse planes during gait with and without load in NCLBP.
4. Core stabilization exercise therapy increases the between-trial variability and peak displacement of the lumbar relative to the pelvis in the sagittal, frontal and transverse planes during gait with and without load in NCLBP.

## Methods

### Study aims

The primary aim of this study is to evaluate the effect of 16-sessions core stabilization program on pain, disability and the spine kinematics while subjects walking with self-selected speed in subjects with NCLBP and comparison with healthy subjects.

### Study design

This study will be conducted at the motion analyzing laboratory of the rehabilitation faculty of Iran University of medical sciences (IUMS). The study is a clinical trial designed to investigate the effect of 16 sessions core stabilization exercise on the trunk and lumbar kinematics relative to the pelvis with and without load during gait and compared with a healthy participants matched group.

### Approval of study protocol

This study has been approved by Ethical committee at Iran University of Medical Sciences (Ethical Approval Number: IR.IUMS.REC 1395.9211342205), (IRCT number: IRCT2016080829264N1).

### Participants

As there is no previous work to estimate the effect size for the kinematic variables considered here, we will conduct a pilot study to compute and estimate the effect size. Then, using power analysis, the required sample size with a power of 0.8 and an α of 0.05 will be determined. Assuming a large effect size, we anticipate recruiting 30 participants (15 in each group) for the study. Both males and females will be recruited in the study. The enrolment will be continued to reach the required sample size. Table 1 summarizes the inclusion and exclusion criteria for the CLBP group. Since the lumbar and trunk kinematics were not different during gait in the movement-based subgroups of NCLBP in a recent study [8], therefore in this study we will examined the NCLBP group regardless of their movement-based classification. Fifteen Healthy non-athlete subjects with no previous history of LBP will be included. Each healthy subject will be matched to a subject with LBP based on the age, sex, height, weight and BMI. In this group, subjects with a history of fracture, trauma and, pain of any other joints in the past also will be excluded from the study. In both groups participants will be excluded from the study if they have BMI higher than 30. Harsted et al. reported that CLBP with a BMI higher than 30 exhibited less variable and lower mean frontal plane range of motion compared to participants with a BMI below 30 [46]. However, less variability of movement of the trunk in this group is generally dependent to larger body mass instead of spine function [46].

**Table 1:** Inclusion and exclusion criteria for the CLBP

Inclusion criteria	Exclusion criteria
The age range of 18–45 years. Current nonspecific Low back pain Lasting more than 12 weeks.	Sciatic nerve root involvement Pain radiating to the leg below the knee Paresthesia in the feet Motor deficits in the lower extremity muscles Acute spinal disc herniation Previous surgery or fractures in lumbar spine or Other bonny structures Neurologic disorders Recent any significant trauma to the musculoskeletal system such as bonny, muscular, ligamentous and soft tissue structures in the upper, lower extremity, head, neck and trunk that might interfere with gait [10, 16] Any clinical conditions such as musculoskeletal, neurological, cardiac or pulmonary or symptom that might interfere with gait [10, 63] Osteoarthritis of the knee and hip [10] Recent unexplained weight loss BMI above 30 kg/m^2^ [46] (see the participants section) Any medical condition which have contraindications to exercise therapy including acute (not reoccurring) low back injury occurring within the last 12 weeks, current tension sign, pregnancy, inflammatory disease, sever osteoporosis, arthritis and bone disease [37, 45, 64]

### Recruitment

The physiotherapist (RB) who is a doctoral candidate with more than 10 years of clinical experience will recruit participants in this study from the IUMS orthopedics and physiotherapy clinics who diagnosed with NCLBP. The physician will refer participants with CLBP to these clinics. In addition, CLBP will be identified and recruited by posters, emails and word of mouth from the university and the surrounding locality. Healthy Participants will be matched in age, gender, body mass index with no history of low back pain. Healthy participants will be recruited through advertisements on bulletin boards and verbal requests in the rehabilitation department at IUMS and surrounding locality.

### Informed consent

On meeting, all of the inclusion criteria in the clinical examination, Participants will be informed about the purpose of the study and the examination and treatment procedures involved in this project and a written informed consent will be obtained from all participants who agree to take part in this study, before data collection. Participants will have the right to refuse core training treatment and withdraw from the study at any time without penalty. The same physiotherapist who is administering the intervention will obtain it.

### Examiner

Participants will be evaluated by a physical therapist who is a PhD candidate with more than 10 years of clinical experience (RB), the same physiotherapist collect the pre-treatment and post-treatment data from each participant and also will provide core stabilization program.

### Equipment

#### Three dimensional motion analyses

A three dimensional approach will be used to kinematic analysis of trunk, lumbar and pelvis pre/post treatment in all participants. Six cameras with the resolution of 1.3 megapixel (Qualisys, Gothenburg, Sweden) will be set up for measure kinematics of the trunk, lumbar and pelvis during gait. Gait kinematic variables will be captured at 100 Hz using a 10MX T20 camera three dimensional motion capture system (Qualisys, Gothenburg, Sweden) and will be analyzed using QTM software (Qualisys, Sweden), MATLAB version R2015 and Microsoft Excel version 2015 (Microsoft, Reading). Using palpation, anatomical landmarks will be identified by physical therapist for each subject and 13 reflective markers with the size of 10 × 8 mm and circular cross-section will be placed on the landmarks to capture kinematics of each segment with the movement analysis system. While the participant are in a flexed standing position supporting themselves upon a stool, marker locations will be confirmed by counting of the spinous processes from T_12_ down to the S_2_ spinous process [3]. The standardized marker location is chosen based on rationales including higher repeatability and maximize visibility as suggested by Seay et al. [18, 47] and Hidalgo et al. [48]. Trunk, lumbar and pelvis segments are defined according to the following to measure trunk and lumbar motion relative to the pelvis:

Trunk segment: is defined by markers on the acromion processes and spinous process of T12 withmotion measured as the relative angle between the trunk and pelvic segments [18, 47].

Lumbar segment: is defined by markers on the spinous process of T12 and two paraspinal markers over the angles of the ribs lateral to T12 (where the transverse process of T12 joins with the rib), with motion measured as the relative angle between the lumbar and pelvic segments [49]. Although, the potential limitations of using paraspinal markers vs. rigid clusters (e.g., soft tissue artefact), this model has been demonstrated high repeatable for detecting lumbar segment movements in CLBP [49, 50]. Kiernan et al. [51] compared two kinematics protocols for quantifying lumbar movement including rigid cluster and skin markers. They reported that rigid clusters might be more susceptible to wobble error relative to the skin markers. Therefore greater levels of variability recorded by the rigid cluster during lumbar rotation suggests the skin surface markers may be more suited to studies where axial rotation is a consideration such as gait studies [51].

Pelvis segment: is modelled by placing 4 markers on the anterior superior iliac spine (ASIS) and posterior superior iliac spine (PSIS) in both sides based on previous studies [48, 52].

In order to detect gait cycle during gait trials 4 markers will be placed on posterior calcaneal tubercle (posterior heel center) and on 5th metatarsophalangeal joint (MTP) of both feet [43].

Data will be recorded for 3 gait trials in both with and without load in pre- and post-intervention. After a static trial, participants will be asked to walk barefoot from one end of a marked walkway to the other that is 10 m in length at their self-selected comfortable walking speed.

### Procedure

Before any intervention and after obtaining the informed consent form, baseline characteristic of participants (such as weight, height) will be measured, and then all of participants will be asked to take the warm up with 5 min of walking in the examination day. Examination will be performed in morning from 9 PM to 12 PM. The reflective markers are attached to the relative anatomic landmarks and participants will stand on the starting point then will performed free self-selected speed walking trials for 6 times, across a 10-m walkway. In order to avoiding the head rotation during walking, a visual target will set on the front of the walkway. A box with maximum 10% of body weight will anteriorly be used to load carrying trials [21]. The box is held with both hands while elbow flexed to 90 degrees, forearms are in mid pronation, wrists are in neutral and arms are beside the body. Participant will be instructed to walking with command, “START”. Then they walk so that they reach to the end point of walkway. Participants repeat each trials 3 times. After a few minutes rest, they started the trials with load.

### Program for core stability training

After the examination day, subjects with NCLBP will participate in the core stabilization program training. Initially, the anatomy and function of local back stabilizing muscles and the way they could be activated will be taught to the subjects. Training sessions will be performed on Saturday, Monday and Wednesday. In the beginning, each participant will perform a warm-up period consisted of stationary bicycle and stretching exercise for 10–15 min. These warm up program include: long sitting position and forward lining to the foot in order to stretching of the hamstring and gastrosoleus muscle, single and double knee to chest from supine position, spinal flexion-extension from 4-point kneeling position, side bending in standing position with and without contralateral arm elevation, Hip flexors stretch from the Thomas test position. According to the previous recommendations, a staged approach will be administered to the stabilization exercise program [53]. This program is designed over 16 individual sessions during 6 weeks. The exercises based on isometric activation of low back, abdominal and pelvis muscles and will be initiated with learning of abdominals, pelvic floor, diaphragm and multifidus muscles to contract isometrically in low load and repetition until their capacity to control the pelvic and lumbar movement are improved. Then the subjects will be learned abdominal hollowing/bracing (co-contraction) in a variety of postures and functional tasks: sitting, quadruped, standing, supine, kneeling, and prone, as well as different degrees of inclination to control loading/gravity. To ensure correct activation of the transversus abdominis muscle, it will be emphasized to the participants that the lower part of the anterior abdominal wall below the umbilical level would be needed to be “drawn in” with the action of this muscle. Also, bulging of the multifidus muscle will be needed to be felt under the therapist’s fingers when they will be placed on either side of the spinous processes of lumbar vertebrae, directly over the belly of this muscle [54]. Progressively participants will instruct to increase holding time and repetition of contraction up to 10 contraction repetitions × 10-s duration [53]. In the first session, the therapist will clearly instruct the patient on how to preferentially activate these muscles lasting about 30 to 45 min. In order to instruct the patient to contract the multifidus during exercise, physiotherapist will provide his fingers on the spinous process of L_4_-L_5_ directly over the muscle belly to felt bulging of the muscle. All training sessions will take place in the same location.

### Outcome measures

#### Pain and disability

Pain intensity will be assessed using a 0–100 visual analog scale (VAS), with 0 representing no pain and 100 representing the worst imaginable pain. Functional disability will be assessed by a Persian version of Oswestry Disability Index (ODI). The development of the ODI was initiated by John O’Brien in 1976 [55]. An ODI can be scored from the eight sections. For each section of six statements the total score is 5 [55]. Validity and reliability of the persian translated version of the ODI has been evaluated by Mousavi et al. and Baradaran et al. [56, 57]. ICC for individual items ranged from 0.43 to 0.80 [56] and 0.91 [57]. The Cronbach-alpha for the ODI was 0.69 [56] and 0.75 [57] showing good internal consistency in both studies.

#### Kinematic analysis

The variability of angular kinematics of the trunk and lumbar spine relative to the pelvic segment is primary interest of this study. A 3D kinematic measurement will be employed to verify the variability of trunk segment in 3 planes of movement relative to the pelvic segment. Previous studies have utilized the Winter’s coefficient of variation (CV) [58] to examine the consistency of movement patterns using the ensemble average of the raw waveforms of repeated trials [6, 15]. A new method of differentiating between the pattern and offset variability has been recently suggested. A large mean offset value effectively deflates the value calculated for variability using the CV [13]. Because of this, O’Dwyer et al. (2009) have suggested the use of methods to differentiate the offset from calculation of the variability in the waveform pattern, the latter being better representative of motor performance repeatability whereas the offset incorporates a greater degree of other variance sources (i.e. marker error). The offset values may also be affected by the reference posture that subjects adopt in each measurement session. Measurement of the reference position may be influenced by the identification of anatomical landmarks and joint centers of rotation, by the markers used, their configuration and their placement on anatomical landmarks. In contrast to offset variability (CVo), waveform pattern variability (CVp) is more manifestly distinguished by the repeatability of the motor performance and is less subject to factors related to reference position [3, 13, 45]. Furthermore, the between trial variability using CVp and CVo will be calculated for the trunk and the lumbar spine kinematics relative to the pelvis across planes of movement. CVp and CVo will be calculated using equations from O’Dwyer et al. [11]. In addition, peak displacement of the trunk movement in three planes will be calculated as the relative angles between the trunk and pelvic segments in three planes (i.e. frontal, sagittal and, transverse). Peak displacement of the lumbar segment will be calculated as the relative angles between the lumbar and pelvic segments in three planes (i.e. frontal, sagittal and, transverse) as described by Crosbie et al. [49].

Data will be normalized to 100% gait cycle and peak values for trunk and lumbar joints angle displacement and will be computed for each cycles and averaged for each subjects. Gait cycle will be considered to initial right heel contact (0%) and subsequent right heel contact (100%) and Heel contacts will be identified as the lowest vertical displacement of a right heel marker [3, 7].

#### Statistical analysis

Data will be analyzed using SPSS version 21 (SPSS Inc., Chicago, IL) software. The normal distribution of data will be analyzed by a Kolmogorov-Smirnov Z test. If the distribution is normal, the outcomes of the studied measurements will be evaluated the absolute change from pre- to post- for VAS, ODI and the kinematic variables using a paired T-test in CLBP group. To determine homogeneity of variances (i.e., variances approximately equal across groups), Levene’s test will be employed. In addition, to evaluate the kinematics variables between CLBP and healthy group, an independent samples T-test will be used to reveal any difference between two groups. A non-parametric test will be used if the data are not normally distributed to compare across two groups (healthy and CLBP) using a Mann Whitney U exact test and, a Wilcoxon signed rank exact test will be used to compare of the variables in pre and post intervention in CLBP. A *p*-value of less than 0.05 will be considered statistically significant. According to the Raymond et al. study [59] minimal change that can be considered with perceived pain questionnaire and ODI in low back pain are clinically useful for interpretation of results. Results of VAS and ODI will be compared with range of minimal important change (MIC) value for pain (15.0–20.0 points) and ODI (10.0–12.0 points) [59].

## Results

Demographic characteristics of participants will be illustrated in the first table. Duration of LBP, VAS and ODI will be included in the Table 2 for the NCLBP group. Other results will be reported in separate tables.

**Table 2:** Demographic and clinical characteristics of study participants

	group		mean ± SD	*P* value
Gender	CLBP	Male		
		Female		
	Healthy	Male		
		Female		
Age (years)	CLBP			
	Healthy			
Weight (kg)	CLBP			
	Healthy			
Height (cm)	CLBP			
	Healthy			
BMI (kg/m2	CLBP			
	Healthy			
	mean ± SD			
Duration of LBP (month) VAS ODI	CLBP			

Values are mean ± standard deviation

Significant set at *P* ≤ 0.05

## Discussion

The present study will investigate the effect of core stabilization exercise on the trunk, lumbar and pelvis kinematic in NCLBP during gait while carrying a load by hands. Based on the previous studies, gait deficiencies can be a symptom following CLBP [3, 8, 45]. Some researchers reported that people with CLBP exhibit less sagittal and transverse plane coordination variability of trunk-pelvis compared to healthy [10, 16–18, 20, 60], other finding revealed that the variability of lumbar kinematic during treadmill walking increases in CLBP [6, 15]. A potential link exists between changes in the trunk and lumbar spine movement variability during gait and core muscles dysfunction [9, 19, 30]. Although, the kinematic changes following LBP have been investigated in several studies [6, 9, 15–18, 20], however there is a lack of study that evaluated the effect of core stability exercise on the trunk, lumbar and pelvis kinematic during gait. To the best of our knowledge, only one pilot study has evaluated the effects of trunk strength and stability training program on spine kinematic [43]. However, they did not determine the movement variability. Core stabilization exercise is the most frequent therapeutic regimen for physical therapy in patients suffering from NCLBP [35, 39, 61, 62]. In addition, no studies have evaluated the effects of core training on gait kinematic during an anterior load carriage task that can challenged the trunk kinematic.

The main result of this study will explore and discuss about the question of what is the effect of core training program on the movement variability and displacement in trunk and lumbar spine relative to the pelvis in NCLBP, specially the transverse plane movement variability of the trunk relative to the pelvis can be altered or no?

The authors also will discuss about the effect of hand-held load on the trunk, lumbar spine and pelvis kinematic during gait before and after the core stabilization training program.

The strength of our study is the measurement of between-trial variability of the trunk and lumbar kinematic (CV_o_ and CV_p_) that is better representation of repeatability of motor performance [3].

Our study will has some limitations, the main limitation of this study is that young and middle-aged NCLBP participants will be included, thereby, the generalizability is limited. In addition, haven’t a control LBP group and the lack of follow-up measurements after the postintervention measurements are other limitations in our study.

## Conclusion

The result of the present study will show the effect of a 16-sessions core training program on the kinematics measures including between-trial variability and peak displacement of the trunk and lumbar spine relative to the pelvic during gait in the NCLBP and healthy. However, we will also evaluate the effect of core training on the mentioned measures while an anterior load carriage task.
